# Tuberculosis1Read before the thirty-second annual meeting of the U. S. Veterinary Medical Association, Des Moines, Iowa, September, 1895.

**Published:** 1895-12

**Authors:** W. B. Niles

**Affiliations:** Veterinary Department, Iowa State College


### THE JOURNAL
OF
### COMPARATIVE MEDICINE AND VETERINARY ARCHIVES.

VOL. XVI. *DECEMBER*, 1895. NO. 12.

### TUBERCULOSIS.[1]

BY W. B. NILES, D.V.M.,
VETERINARY DEPARTMENT, IOWA STATE COLLEGE.

THE subject of tuberculosis is such an extensive one and has been so many times discussed before this body that I shall limit myself in this paper to a consideration of certain features only, dealing principally with the most prominent points observed in our experiment-station work here in this State.

During several years we have heard much regarding the prevalence of bovine tuberculosis in the East. A number of years ago we read that in and near the large cities a large percentage of the milch-cows were tuberculous. The impression was given that the way in which the animals were kept was largely accountable for the great number of cases, and that away from the cities where the conditions favorable to the spread of the disease did not prevail it was not very prevalent. Later, when the tuberculin-test was first applied to the Pennsylvania College herd and a few others in Pennsylvania and New York, with the result that the previous statements concerning the great prevalence of the disease were much more than confirmed, we still looked upon the disease as mainly one of the East and not of much concern to the people of the West. Our agricultural and live-stock papers often referred to the disease as occurring frequently in the East and very rarely in the West. This opinion was held by a great majority of our people until very recently. Veterinarians being scarce, and not well acquainted

[1] Read before the thirty-second annual meeting of the U. S. Veterinary Medical Association, Des Moines, Iowa, September, 1895.

with the disease, what cases did occur largely escaped observation.

To a few veterinarians, however, tuberculosis in the West is by no means new. Our State Veterinarian, Dr. Stalker, observed cases in some of the breeding-herds twenty-five years ago. The writer ten years ago saw several cases in a valuable herd of short-horns. During recent years Western veterinarians have from time to time recognized cases in different sections of the country, but having to rely entirely on a physical examination as a means of diagnosis but little was learned regarding the extent of the invasion. It was observed, however, that breeding-herds seemed most involved, and that in spite of efforts to stamp it out the affection lingered in the herd, one or more animals dying yearly.

About a year and a half ago the veterinary section of our State Experiment Station began a systematic study of the disease in this State, using tuberculin as an aid in diagnosis. The work was undertaken for the purpose of determining the prevalence of the disease in the State, and determining in conjunction with other workers the value of tuberculin as a diagnostic agent. Our observations have extended to all classes of cattle, viz., breeding-herds, dairy-herds, and private cows, as well as to animals of all the prevailing breeds. As the observations have been largely made on herds suspected of being diseased, the results do not give us a very definite idea of the percentage of tuberculous animals in the State; yet much has been learned regarding the occurrence of the disease under conditions existing in this section of the country.

Breeding-herds have been found involved to a considerable extent. Several herds have been found diseased, and in some a very large percentage affected. A test of thirteen animals of an Aberdeen Angus herd of the World's Fair winners showed six of them tuberculous. Eight of the thirteen were apparently in the best of health, and were included in the test to show the owner that a reaction would not occur on healthy animals. Of these two, or 25 per cent., reacted. Previous deaths from tuberculosis and a post-mortem examination on two of the reacting animals confirmed the test in this instance. That a test of the entire herd would have shown a large percentage affected cannot be questioned.

A test of a farm-herd containing full-blood short-horn cattle lately purchased from three different breeders showed tuberculosis in animals coming from each herd. All were bought in apparent health, and so appeared at the time of the test. Out of seven coming from one herd, two, or about 28 per cent., reacted. I have since learned that the owner of this herd has for some time been trying to get rid of the disease by the weeding-out process. From information derived from different sources the disease is known to exist in several other breeding-herds. Thus we learn that while all our breeding-herds are not affected many of them are, and some of them badly so.

In dairy-herds the test has shown similar results. In a herd of fifty cows twenty-seven showed characteristic reactions. About ten others gave a rise of between one and two degrees. A slaughter of the latter with those which did not react, under circumstances that did not permit a very careful examination, revealed eleven tubercular carcasses. Counting these with the twenty-seven showing positive reactions, we have thirty-eight out of the fifty tuberculous. The testing of this herd led to the examination of almost all the dairy-cattle supplying milk to the city of Waterloo. About three hundred and twenty-five animals were examined, including the herd just mentioned. Of these thirty-eight, or about 11 per cent., gave the characteristic reaction. Three of the herds were badly affected, and contained most of the cases found. The percentage would have been a little larger had not all the visibly diseased in one herd been culled out and sold before the test was made.

In another county two dairy-herds, consisting of eighty-four animals, when tested, showed twenty-three positive reactions, all occurring in one herd. This herd was not suspected of being diseased, and a case of tuberculosis could not have been positively diagnosed at the time by physical examination alone. A test of another dairy-herd, also supposed free from disease, revealed a similar state of affairs, twelve out of twenty reacting.

One of the most interesting herds tested was one in Western Iowa. This herd had been on the farm for a considerable time, most of the animals having been raised by the owner. With the exception of four or five cows the herd had never been kept within a barn or shed of any kind, but were kept much in the same way as cattle on the Western plains, the only source of profit being the increase. Six cows had died in the herd during the summer preceding the test, which was made in December. At this time the herd consisted of thirty-five head, ranging in age from a few months to ten years. The test resulted in ten reactions on animals of different ages out of the thirty-five tested. As will be noticed later, those reacting showed in every case well-marked tubercular lesions. Other herds might be cited, but a sufficient number have been mentioned to show that the disease is by no means rare in this State. Of the total number tested by the Station and State veterinary authorities, over 10 per cent. have reacted to the test. This is, of course, too high for the general average, as many of the herds tested were suspected or known to be diseased. We can say definitely, however, that the disease is quite prevalent; that more diseased herds exist, and that these contain more affected animals than any of us supposed before beginning the work with tuberculin. The conditions in many other Western States are no doubt much the same as here.

*Predisposing causes.* What are the predisposing factors, and to what extent do they influence the spread of the disease? This is an important question, and one often asked. Let us consider it briefly. Most writers on the subject of tuberculosis lay great stress on predisposing causes, emphasizing in-and-in breeding, the keeping of many animals together in imperfectly ventilated quarters, excessive drain upon the system by stimulating milk-production, and some others as being prominent. Many agriculturists go so far as to say that the careful breeder who breeds to secure vigorous and hardy animals and looks carefully to the construction of his buildings has little to fear from tuberculosis. We do not doubt that these factors may play an important part in spreading the disease, but we are firmly of the opinion that their importance has been much overrated.

A majority of the affected herds observed in this State *have not been kept in poorly ventilated quarters and were not herds that* had been bred and kept in such a way as to lower the vitality of the animals. For example, our breeding-herds, in almost every instance, receive the very best of care, and are not kept in what would be considered close quarters. Still, as I have shown, many of them are affected, and in some cases a *large* percentage of the herd. Neither does the breed seem to play an important part, for we have found the disease in Angus, short-horns, Jerseys, and scrubs. In the affected dairy-herds, in no instance was the diseased herd one that had been bred solely for milk-production, and in but one case was the herd kept in unventilated quarters. As a rule, the buildings in which the affected herds had been kept would be considered open enough for ample ventilation. It should be remembered in connection with this part of the subject that cattle in this section are, as a rule, kept indoors but a small part of the time. In summer they are not stabled at all or only long enough to be milked, and in winter are only stabled over night and on stormy days.

In the herd that had never been stabled (already referred to) we have an example of a badly-infected herd that had not been subjected to any of the conditions usually considered necessary in causing extension of the disease. Yet we find that, counting the six that had recently died, almost 29 per cent. were diseased. The herd had not been inbred, was not predisposed by age, was not kept and never had been (with the exception of four or five cows) in a building of any kind, and were only predisposed by being subjected to the inclemencies of the weather and possible scarcity of food during the drouth of summer. The observations on this herd show that the disease *will spread* where animals only come in contact in the yard or field, and it seems to me that our observations on the disease in this State point very conclusively to the fact that bad ventilation, breed of animals, and other predisposing factors are not as much concerned in the production of the disease as has been generally supposed. I think we should not lose sight of the fact, and the breeder should be made to understand, that if the disease is once introduced into the herd, if a diseased animal in any way cohabits with the others, that the affection will spread no matter what the sanitary conditions may be.

*Reliability of tuberculin in diagnosis.* So much has been written about the use of tuberculin as an aid to diagnosis that I shall not dwell long on this point. In the herds I have personally examined but a very small percentage of the cases could be detected by a physical examination alone. To us, and as far as known by the owner, a majority appeared in perfect health. In a few instances emaciation, cough, and abnormal lung-sounds could be easily detected. Without tuberculin not a case of tuberculosis could have been detected in some herds that by its use were shown badly infected. This coincides with the observations of others. With regard to the reliability of the test, our results are similar to those reported by all who have given the agent an extended trial. We have post-mortem records on over fifty cattle, and in but one instance has a postmortem on an animal showing a characteristic reaction failed to reveal tuberculosis. In the one case no naked-eye lesions could be found, but sections of the bronchial gland under the microscope showed small tubercular areas. Where a characteristic reaction occurs, that is, where there is a marked rise above the normal, continuing for several hours, I believe in every case the animal has tuberculosis, whether the post-mortem examination shows the lesions or not. That is, I would place a greater reliance upon the tuberculin-test than I would on the post-mortem examination. We all know that the lesion may be easily overlooked, or microscopic, and thus escape detection.

*The amount of rise in temperature.* As to what constitutes a reaction in every case is a question not easily answered. In many diseased herds there are a few animals that show a rise of between one and two degrees above the normal, or what we consider the normal temperature. When the rise is due to the reaction of the tuberculin, I consider even a rise of one degree a reaction; but the uncertain factor is the normal temperature. It is difficult to determine whether the slight rise may not be due to other causes and not to the tuberculin. For this reason great care and familiarity with all the details of the test are necessary in order to avoid errors. We have found the temperature of cattle to vary greatly. It may vary three degrees during the twenty-four hours, and the cow be in perfect health. In the winter the 8, 9, and 10 o'clock temperature in most herds is very low, and is the minimum temperature secured. In the morning, if the cattle are in the barn, the temperature is high and reaches the maximum during the forenoon. If turned out and watered the temperature immediately falls, often as much as two degrees. In summer, on a hot night, I have found the 8 P.M. temperature very high, reaching two or three degrees above the minimum. As a rule, in summer the evening temperature is the highest. Thus we see that the natural temperature of cattle is by no means constant, and that a rise of one or two degrees may occur after injecting and not indicate a reaction in the least. The operator must, by noting the temperature of the entire herd and by considering the conditions surrounding the test, determine how much of the rise is due to the tuberculin and how much to other causes. As opportunity seldom offers for the slaughter and examination of animals showing only a slight rise, we cannot easily settle the amount of rise necessary to constitute a reaction or determine how many tuberculous cattle fail to react. In our work a slaughter of ten animals, showing a rise of less than two degrees, showed almost all affected. In some other cases showing slight rise, as follows: Animal No. 1, 1.8°; No. 2, 1.8°; No. 3, 1.6°; No. 4, 1.4°; No. 5, 1.3°—the post-mortem examination showed tubercular lesions in every case. We may remark that there are two kinds of cases showing the slight rise sufficient to render the animal suspicious, viz., those that are so badly diseased that a physical examination will detect them, and those apparently healthy. I have observed two or three of the former and a considerable number of the latter. When the tuberculin indication can be confirmed by a physical examination there is no trouble; but when the only indication of disease is the slight rise in temperature the results are unsatisfactory. To repeat, I believe with others who have expressed an opinion on this point that a very slight rise due to the action of tuberculin, and continuing for a few hours, indicates tuberculosis.

The duration of the reaction and the time of the maximum temperature, while quite constant, vary much in a few cases. Usually the rise begins in from ten to twelve hours and reaches the maximum in from fifteen to eighteen hours. In a majority of the cases under observation this maximum has been reached in about fifteen hours. In a few cases this has occurred at a much later time, and would have escaped detection had not the temperature been observed well into the afternoon. To illustrate, in one animal the rise did not begin until after the fourteenth hour, and did not reach the maximum until the twentieth hour. In another case the reaction began at the sixteenth hour, reaching the maximum twenty-one hours after injection. This case could easily have escaped detection. Both were plainly tuberculous on post-mortem examination.

In our work the exceedingly small dose of tuberculin, recommended by Dr. Kinnell in the *Veterinary Magazine*, has not been tried. The amount used in most cases has been two c.c. of American lymph for the ordinary-sized animal. In a few cases the dose has been lessened one-third without apparent change in the results. As it seems to me of advantage to detect every case in the herd if possible, I fail to see the advantage of the minute dose, except as a saving of material, which would be outweighed by the advantages of the larger dose. In our hands the product furnished by the Bureau of Animal Industry, the American Hygienic Company, and that prepared in Germany, gave like results. In practice I prefer that made here, as it needs no dilution and can be used as received.

The effects of a subsequent injection or injections is an important problem. If the first test shows the animal suspicious, can we clear up the mystery by testing the patient again? If this question could be unqualifiedly answered in the affirmative the doubtful animals in the herd would give us little concern. Unfortunately this is not the case. Observations by different ones show that in a majority of cases the rise in temperature following the first injection is greater than in subsequent ones. In glancing over a report of the Bureau I find that, out of forty-five animals injected twice, nine showed a higher rise on second injection and thirty-six a lessened reaction, and that eleven of these did not react at all. In the New Jersey station herd three animals showed greater rise, and nine—four of which did not react at all—a less rise. The Pennsylvania and Virginia herds showed like results.

In our work two Holstein cows that showed following first injection a maximum temperature of 106.6° and 105.2°, respectively, revealed on second test a maximum of 105.6° and 103.8°. On third injection, several weeks later, there was no appreciable rise. An Angus cow showing a maximum at first of 105.6° dropped to 102° on second and 103.4° on the third test. A post-mortem on the Holsteins revealed a bronchial gland, plainly tuberculous in each. In one only a few small tubercles were present, but in the other the gland was much enlarged and contained several large tubercles in advanced stages of degeneration. A dairy-cow giving a rise of over two degrees on first test showed but a very little rise when tested three months later. A post-mortem revealed only a few calcified nodules in one bronchial gland. A second injection three months after the first on four short-horn cows showed in each case a slightly higher rise than at first. The amount of tuberculin used in the second test was slightly larger, which may account for the second reaction being greater than the first.

During the summer we have at intervals been injecting four animals for the purpose of learning the effect of repeated injections. Two of these ceased to react after the second injection, one after the fourth, and the other after the fifth. All showed a less rise after the second injection. Four others that have recently received a second injection with slight increase in size of the dose five months after the first have also shown a less rise than that first revealed. I think it can safely be said that the results of the first injection are, as a rule, the most satisfactory, but if the dose be considerably increased and several weeks elapse between tests we believe a second injection can usually be relied upon.

The effect of tuberculin on healthy animals may be considered briefly. It has been claimed by some of the objectors to the test that the agent is injurious to animals in health and may even cause tuberculosis. The last opinion is not held by anyone familiar with the preparation and the way in which it is prepared. The experiment of Prof. Law and the observations of others go to prove that the injection of tuberculin on the healthy animal is followed by no bad results whatever. Our observations are thoroughly in accord with the above. Healthy cattle seem to suffer no inconvenience. Some dairymen say that there is a slight falling off in the milk-supply, but this is only temporary, and is no doubt largely due to the changed conditions under which the animals are kept during the test. The influence of the injection on the course of the disease is a very important question to consider. The fact that it has been assumed by some to hasten the course of the disease, causing a mild case to become acute, has been heard of by the objectors to the use of tuberculin, and some are trying to make a good deal of capital out of the statement. Does the use of tuberculin tend to hasten the progress of the disease? It seems to me that a sufficient number of observations have been made in different parts of the country so that this question can be partially answered. The results of the work here do not go to show that the mild case is made worse. In no case under observation has a rapid decline followed the use of the agent. Whether the opposite may occur is not easily determined. In the two Holsteins that received the three injections there does not seem to have been either decline or improvement, but as we cannot determine in these mild cases the extent of glandular lesions at the time of the test we cannot tell, when the post-mortem is held some time later, whether slight improvement or a change for the worse has occurred. In the short-horn animal referred to, in which the tubercles were found calcified three months after the first injection, there were certainly no bad results, but, on the contrary, indications of improvement.

In our work a great majority of the cases observed have been of a mild type. In these mild cases the animal appears in perfect health, often fat and sleek, giving a good flow of milk. It may be worthy of note that the animal may show very marked tuberculosis of the lungs and pleura as well as of other organs, and still remain in average flesh and appear to the owner as in good health. As many herds containing a large percentage of tuberculous animals show no acute cases, the mild or latent cases must communicate the disease, and we believe the animal with the disease in any form should always be considered a source of danger. Concerning the progress of the disease in these mild cases much remains to be learned. It certainly progresses very slowly toward a fatal termination when the animals are well fed and otherwise well cared for. How many recover by the tubercles becoming calcified and encysted is at present a matter of conjecture only. We have observed that in diseased herds, poorly fed in winter or running on poor pasture in summer, the disease progressed apparently quite rapidly, as in some herds examined several deaths had occurred, and at the time of examination many animals were quite badly diseased. I do not wish to convey the impression that this kind of care is necessary to hasten the progress of the disease to a fatal termination. Such is not the case. Some of the worst cases seen were in a herd of World's Fair winners, animals that had always received plenty of nutritious food and kept in well-ventilated quarters, etc.

In a majority of the cases examined after death the lesions have not been extensive. Very often they are confined to one mediastinal or bronchial gland. Some show one or more tubercular abscesses in the lungs, while they may be so small as to escape detection unless the organs are very carefully examined. In one case the only evidence of tubercular lesions was a group of tubercles the size of a silver quarter on the peritoneum in the region of the flank, and an inflamed area on the spleen where it came in contact with the tubercular spot. Several have shown great enlargement of the post-pharyngeal glands, with tubercular abscess in centre. Five per cent. or over have shown the lesions in the udder. A small percentage have shown very extensive lesions of lungs and pleura, as well as tubercles in other regions. Occasionally one in which a very careful physical examination before death was necessary to detect the disease has proven quite badly diseased on post-mortem examination. These cases illustrate how misleading a physical examination may be.

As this paper is already of sufficient length I will not discuss other points, and will only repeat that the work in this State shows that the disease is quite prevalent in the West; that all breeds and classes of cattle are affected; that the mild form of the disease is that most often seen; and that, although existing in the mild form, it will spread where cattle are well cared for and not subject to the causes usually considered necessary in order for the affection to spread and attack a large percentage of the herd. Also that in tuberculin we have an agent that can be relied upon in the diagnosis of the disease, the mistakes accredited to it by some having very probably been due to the carelessness of the operator.

Hoping that this may serve to open a discussion of this subject which will bring out valuable information, I will conclude by extending thanks to all who have so kindly assisted our Station staff of veterinarians in the work. Some of the assistant State veterinarians and our veterinary students have rendered valuable assistance in carrying out the details of the work. To some of the practitioners and to the owners of many of the diseased herds thanks are due for assistance in demonstrating the practicability of the test and the consequent possible eradication of the disease from an affected herd.

